# The role of SLC7A11 in diabetic wound healing: novel insights and new therapeutic strategies

**DOI:** 10.3389/fimmu.2024.1467531

**Published:** 2024-09-03

**Authors:** Wei Zhang, Jiawei Feng, Yiming Ni, Gen Li, Yuqing Wang, Yemin Cao, Mingmei Zhou, Cheng Zhao

**Affiliations:** ^1^ Shanghai Traditional Chinese Medicine Integrated Hospital, Shanghai University of Traditional Chinese Medicine, Shanghai, China; ^2^ Institute of Interdisciplinary Integrative Medicine Research, Shanghai University of Traditional Chinese Medicine, Shanghai, China

**Keywords:** SLC7A11, system Xc-, diabetic wound healing, efferocytosis, oxidative stress, ferroptosis

## Abstract

Diabetic wounds are a severe complication of diabetes, characterized by persistent, non-healing ulcers due to disrupted wound-healing mechanisms in a hyperglycemic environment. Key factors in the pathogenesis of these chronic wounds include unresolved inflammation and antioxidant defense imbalances. The cystine/glutamate antiporter SLC7A11 (xCT) is crucial for cystine import, glutathione production, and antioxidant protection, positioning it as a vital regulator of diabetic wound healing. Recent studies underscore the role of SLC7A11 in modulating immune responses and oxidative stress in diabetic wounds. Moreover, SLC7A11 influences critical processes such as insulin secretion and the mTOR signaling pathway, both of which are implicated in delayed wound healing. This review explores the mechanisms regulating SLC7A11 and its impact on immune response, antioxidant defenses, insulin secretion, and mTOR pathways in diabetic wounds. Additionally, we highlight the current advancements in targeting SLC7A11 for treating related diseases and conceptualize its potential applications and value in diabetic wound treatment strategies, along with the challenges encountered in this context.

## 1 Introduction

Diabetes mellitus (DM) is characterized by persistent hyperglycemia caused by defects in insulin secretion or function, classifying it as a chronic metabolic disease. It is projected that by 2045, 783 million people will be affected (1, 2). Long-term exposure to high blood sugar levels diminishes immune responses and triggers complications related to nerves and blood vessels, leading to conditions such as retinopathy, nephropathy, neuropathy, and diabetic wounds (3). Diabetic foot ulcers (DFUs), a severe and common type of diabetic wound, significantly contribute to morbidity and mortality globally and impose substantial economic burdens. These chronic ulcerative wounds are often exacerbated by tissue ischemia or hypoxia and, due to impaired sensory nerve function, result in a high rate of limb amputations. The estimated mortality associated with DFUs is 5% within one year and 42% within five years (4). Despite ongoing research into the pathophysiological mechanisms and treatment options for diabetic wounds, understanding the explicit mechanisms and effective treatment strategies remains limited.

During the wound healing process, phagocytic cells such as dendritic cells (DCs) efficiently mediate efferocytosis to clear apoptotic cells from the injured area, thus promoting tissue repair and suppressing autoimmune responses (5, 6). They release a plethora of molecules, such as CD86 and C-C motif chemokine receptor 7, facilitating the production of prostaglandin E2 (PGE2), interleukin-1β (IL-1β), and interleukin-10 (IL-10) (7, 8). However, the efferocytosis by macrophages is impaired in diabetes, exacerbating the inflammatory response, leading to delayed wound healing and impaired tissue regeneration (9, 10). Moreover, long-term hyperglycemia in diabetic patients increases mitochondrial reactive oxygen species (ROS) production, inducing oxidative stress and lipid peroxidation, leading to cellular dysfunction and death (11, 12). Impaired iron metabolism in diabetes results in the accumulation of free plasma iron, further inducing ferroptosis. Ferroptosis is a non-apoptotic cell death dependent on ROS and iron availability, proven to impair diabetic wound healing (13, 14). These alterations collectively contribute to inflammation and oxidative stress in the wound area, ultimately affecting wound healing (15).

SLC7A11 (xCT) is the subunit of the cystine/glutamate antagonist system Xc- and plays a vital role in intracellular cystine uptake and glutathione (GSH) synthesis (16). GSH, a major antioxidant in cells, maintains the internal antioxidant balance, which is crucial for combating oxidative stress and ferroptosis in diabetic wound healing (17). The uptake of cystine through SLC7A11-mediated activates the PI3K/AKT signaling pathway, leading to the stimulation of mechanistic target of rapamycin complex 1 (mTORC1), which in turn inhibits lipid peroxidation and ferroptosis (18). This process is also linked to macrophage polarization. Moreover, glutamate exported by system Xc- to the extracellular space reduces insulin secretion (19). Beyond mediating basic system Xc- functions, SLC7A11 regulates efferocytosis by DCs and affects the release of factors such as growth differentiation factor 15 (GDF15) (5). These findings suggest that SLC7A11 may influence wound healing through multiple mechanisms. A thorough understanding of SLC7A11’s regulatory mechanisms will aid in developing effective targeted therapies for diabetic wound treatment.

In this review, we first discussed the key mechanisms that lead to delayed wound healing in diabetes, then introduced in detail the regulation of the SLC7A11-dependent signaling pathway on wound healing in diabetes, and conceptualized its potential application and challenges in diabetes wound treatment strategies. These insights can provide a theoretical basis and new treatment methods for treating diabetes wounds. 

## 2 Overview of SLC7A11

### 2.1 The structure of system Xc- and its transfer function

**Figure 1:**
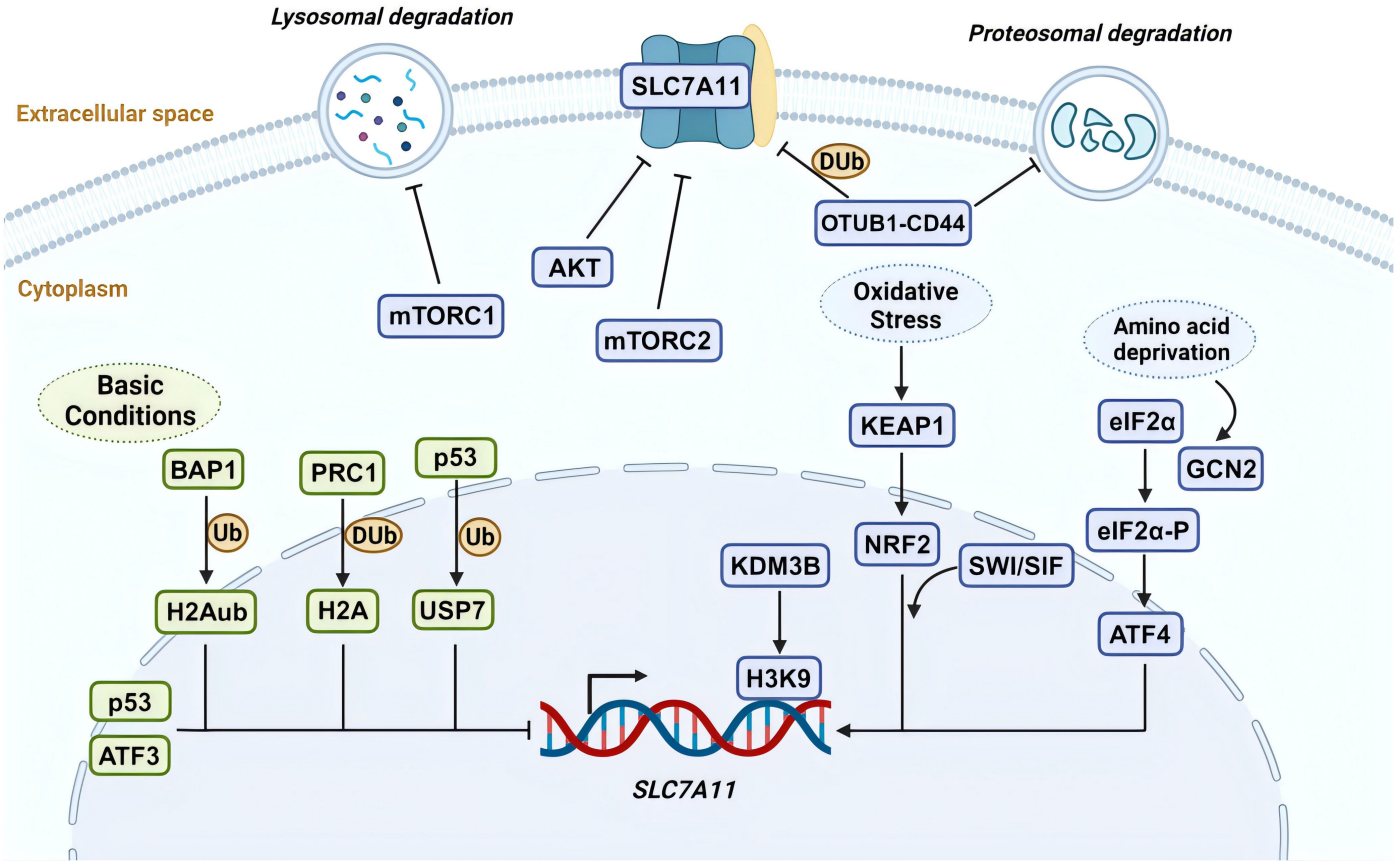
SLC7A11 regulation by various mechanisms. The expression and function of SLC7A11 are regulated at multiple levels under both basal and stress conditions. Under basal conditions, ATF3 and p53 suppress the transcription of SLC7A11. BAP1 inhibits its expression by removing H2Aub ubiquitin from the SLC7A11 promoter, whereas PRC1 ubiquitinates H2A on the SLC7A11 promoter, also suppressing SLC7A11. p53-mediated nuclear translocation of USP7 further inhibits SLC7A11 transcription by reducing H2Bub ubiquitination occupancy. Under stress conditions, ATF4 activates SLC7A11 transcription via the GCN2-eIF2α signaling pathway in response to amino acid deprivation. During oxidative stress, KEAP1-mediated degradation of NRF2 is inhibited, allowing stable NRF2 protein to translocate to the nucleus and activate the transcription of SLC7A11 and other antioxidant genes. Additionally, the SWI/SNF chromatin remodeling complex facilitates NRF2-mediated transcriptional activation of SLC7A11. The H3K9 demethylase KDM3B promotes its transcription by reducing H3K9 methylation at the SLC7A11 promoter. In terms of post-translational regulation, OTUB1 and CD44 form a trimeric complex with SLC7A11, ubiquitinating it to prevent its degradation by the proteasome, thereby stabilizing SLC7A11 protein. High cell density inhibits mechanistic target of rapamycin 1 (mTORC1) and promotes SLC7A11 degradation in lysosomes. mTORC2 and AKT inhibit the activity of the SLC7A11 transporter by phosphorylating serine 26 on SLC7A11. Created with BioRender.com.

system Xc- belongs to the heterodimeric amino acid transporter (HAT) family, comprising the light chain subunit SLC7A11 and the heavy chain subunit SLC3A2 (4F2hc) (20). These two subunits are connected via extracellular covalent disulfide bonds and fulfill distinct roles (21). SLC7A11 is a multi-pass transmembrane protein that facilitates the activity of system Xc- (22), while SLC3A2 is a single-pass transmembrane protein, acting as a stabilizing agent for the stability and proper membrane localization of SLC7A11 (23). system Xc- is situated on the cell membrane and functions as a sodium-independent reverse transporter, expelling internal glutamate and absorbing extracellular cystine at a 1:1 ratio (24, 25). Cysteine is an amino acid involved in protein synthesis, post-translational modifications, and maintaining redox balance (26, 27). It serves as a precursor for antioxidants and biological macromolecules with antioxidant qualities such as GSH (composed of cysteine, glutamate, and glycine), taurine, and hydrogen sulfide (26, 27). The cellular cysteine acquisition pathway involves the synthesis of homocysteine with serine. This process occurs in tissues, including the liver, kidney, pancreas, and specific cell lines in the transsulfuration metabolic pathway (28). However, most cysteine is absorbed from the extracellular environment via the system Xc- (**Figure 1**). Unstable extracellular cysteine rapidly oxidizes to cystine, which the system Xc- transports into the cell. The high reductive environment inside the cell quickly converts cystine back to cysteine.

Cysteine and glutamate combine enzymatically to form γ-glutamylcysteine by glutamate-cysteine ligase. Glutathione synthase adds glycine to γ-glutamylcysteine to form reduced glutathione. This synthesized reduced glutathione acts as a cofactor for ROS detoxification enzymes such as glutathione peroxidase (GPX) and participates in the breakdown of peroxides such as hydrogen peroxide. This process depletes GSH and helps to protect cells from oxidative damage incurred by ROS. Reduced GSH is oxygenated to glutathione disulfide in the presence of GPX. Subsequently, glutathione reductase utilizes NADPH to regenerate reduced GSH from glutathione disulfide (20). As a primary antioxidant in cells, the continuous synthesis and regeneration of reduced GSH are indispensable for maintaining cellular redox homeostasis and mitigating oxidative stress-induced damage (29). This ongoing supply of GSH is particularly crucial in conditions characterized by elevated oxidative stress, such as chronic inflammation or metabolic disorders like diabetes (30). Furthermore, the transport of extracellular cystine into the cell via the system Xc- is critical for sustaining intracellular levels of cysteine and GSH (24).

In the subsequent discussion, we shall now address the extended functions of system Xc-, including roles in oxidative stress, ferroptosis, and immune-inflammatory responses. Given that SLC3A2 also acts as a chaperone for multiple other amino acid transporter proteins and has multifunctional capabilities beyond its role in system Xc- (23). We will concentrate on investigating the impact of SLC7A11 on diabetic wound healing, specifically its regulatory roles in various physiological and pathological mechanisms.

### 2.2 Regulatory mechanisms of SLC7A11

Multiple mechanisms precisely control SLC7A11’s expression and activity to maintain its appropriate function in organisms (**Figure 1**). These include transcriptional regulation by transcription factors, epigenetic regulators, and post-transcriptional regulatory mechanisms such as tuning its mRNA levels, subcellular localization, protein stability, and transporter protein activity (20). In this section, we will explore how various mechanisms regulate SLC7A11 and the downstream biological effects it mediates.

#### 2.2.1 Transcriptional regulation of SLC7A11 by transcriptional factors

SLC7A11 is induced to be expressed under diverse stress conditions, including oxidative stress, amino acid starvation, metabolic stress, and genotoxic stress, contributing to maintaining redox homeostasis and supporting cells alive under stress conditions (20). Activating transcription factor 4 (ATF4) and nuclear factor erythroid 2- related factor 2 (NRF2) are core transcription factors involved in the stress-induced transcription of SLC7A11. ATF4, belonging to the ATF/CREB (activating transcription factor/cyclic AMP response element binding) transcription factors family, is activated primarily through the mRNA translation mechanism under stress conditions, including hypoxia, endoplasmic reticulum stress, amino acid starvation, and viral infection, and facilitates target gene transcription in the nucleus (31). Under non-stress conditions, the translation of ATF4 mRNA is inhibited by upstream open reading frames (uORFs) in its 5′ untranslated region. Under stress conditions, the translation of ATF4 uORF is inhibited, which involves various upstream kinases activating the phosphorylation of eukaryotic initiation factor 2α (eIF2α) under cell stress, leading to an increase in ATF4 protein levels (31). Research indicates the general control non-derepressible-2 (GCN2)-eIF2α-ATF4 signaling pathway is activated during amino acid starvation, upregulating the expression of SLC7A11 (32). This promotes the transcription of genes related to stress response and amino acid metabolism, adapting to the state of amino acid deprivation (32). NRF2 is another key transcription factor in promoting SLC7A11 transcription and mainly mediates oxidative stress-responsive transcription processes (33). Similarly, under non-stress conditions, NRF2 interacts with kelch-like ECH-associated protein-1 (KEAP1). KEAP1 serves as a substrate adapter protein for the Cullin3-dependent ubiquitin ligase complex, promoting NRF2 to be degraded by the proteasome via KEAP1-Cullin3-mediated ubiquitination. When oxidative stress occurs, KEAP1 is inactivated, and its degradative effect on NRF2 is impaired, enhancing the stability of NRF2 protein. Subsequently, NRF2 combines with an antioxidant response element to regulate the transcription of antioxidant defense and redox homeostasis-related genes such as SLC7A11 (34).

Furthermore, the expression of SLC7A11 can also be transcriptionally repressed (16). p53 is recognized as a transcription factor that represses SLC7A11 expression (35). Under ferroptosis-inducing conditions, p53 induces ferroptosis by partially inhibiting the expression of SLC7A11, whereas its absence leads to increased resistance to ferroptosis through the upregulation of SLC7A11 (35). Activating transcription factor 3 (ATF3) is another member of the ATF/CREB transcription factors family. It mainly regulates the transcription of SLC7A11 under basal conditions without affecting the stress-induced expression of SLC7A11. A study confirmed that ATF3 expression is upregulated after erastin (a potent inducer of ferroptosis) treatment (36), binds to the SLC7A11 promoter, and represses its expression in a p53-independent manner and that ATF3 contributes to erastin-induced ferroptosis by downregulating SLC7A11 (37).

In summary, SLC7A11 expression is repressed by ATF3 and p53 under basal conditions, whereas various stress conditions promote SLC7A11 transcription partly via ATF4 or NRF2. Together, these regulatory mechanisms maintain a low-level state of SLC7A11 *in vivo* under normal conditions and are ready to respond to stress stimuli to satisfy physiological demands.

#### 2.2.2 Epigenetic regulation of SLC7A11 transcription

Epigenetic regulation of gene transcription occurs primarily through DNA and DNA-associated histones modifications, such as methylation, phosphorylation, ubiquitination, and acetylation (38). Particular DNA or histone modifications are frequently related to particular gene transcription types (16). SLC7A11 is a critical transcriptional target of the nuclear deubiquitinating enzyme BAP1 (39). BAP1 deubiquitinates histone 2A (H2Aub) on the SLC7A11 promoter and represses SLC7A11 expression in a p53-independent way (39, 40). Polycomb Repressive Complex 1 (PRC1) is the primary ubiquitin ligase mediating H2Aub (41). Similar to BAP1, PRC1 inhibits the expression of SLC7A11, even though PRC1 and BAP1 have opposite effects on the levels of H2Aub on the SLC7A11 promoter. The key to this repression of SLC7A11 may lie in the balance of H2A ubiquitination and deubiquitination mediated by BAP1 and PRC1 instead of changes in the level of H2Aub itself (16). Contrastingly, monoubiquitination of histone 2B (H2Bub) at the lysine 120 site is usually related to transcriptional activation (42). As research indicates, erastin treatment reduces the overall levels of H2Bub and the occupancy of H2Bub on the SLC7A11 promoter, thereby inhibiting SLC7A11 transcription (43). Moreover, the repressive function of histone H3 lysine 9 (H3K9) methylation on transcription has been confirmed (44).

A recent study showed that overexpression of the H3K9 demethylase KDM3B reduces H3K9 methylation and increases SLC7A11 expression by removing repressive marks on the SLC7A11 promoter. This enhances protection against erastin-induced ferroptosis (45). BRD4 belongs to the bromodomains and exogenous structural domains family that may facilitate SLC7A11 transcription via epigenetic mechanisms (46). Recently, the chromatin remodeling activity of the switch/sucrose non-fermentable (SWI/SNF) complex was found to be associated with SLC7A11 transcriptional regulation (47). Specifically, ARID1A, a SWI/SNF complex component, combines with the SLC7A11 promoter to promote NRF2-mediated activation of SLC7A11 transcription (47).

#### 2.2.3 Posttranslational regulation of SLC7A11

Various post-translational mechanisms also regulate the localization, stability, and transporter activity of SLC7A11. OTUB is a deubiquitinase of the Ovarian Tumor family. It was shown that OTUB and adhesion molecule CD44 variant (CD44v) regulates the stability of SLC7A11 and sensitivity to ferroptosis (48). Precisely, SLC7A11, ubiquitin aldehyde binding 1 (OTUB1), and CD44v form a trimeric complex, interacting to maintain cell surface localization and stability of SLC7A11 (49, 50). Furthermore, the transmembrane protein SLC7A11 is also thought to be regulated by lysosomal degradation due to its localization on the plasma membrane (16). The mechanistic target of rapamycin (mTOR) belongs to the PI3K-related kinase family, which is a serine/threonine kinase. mTOR exists in two distinct kinase complexes, mTORC1 and mechanistic target of rapamycin complex 2 (mTORC2), exerting regulatory effects on SLC7A11. Studies have shown that the inactivation of mTORC1 can promote the lysosomal degradation of SLC7A11, even though the specific mechanism remains unknown (51). mTORC2 interacts with SLC7A11 and responds to growth factor stimulation by phosphorylating serine 26 in the SLC7A11 cytoplasmic region, resulting in inhibition of its transporter activity (52).

## 3 Excessive inflammatory and oxidative stress states in diabetic wound healing

**Figure 2:**
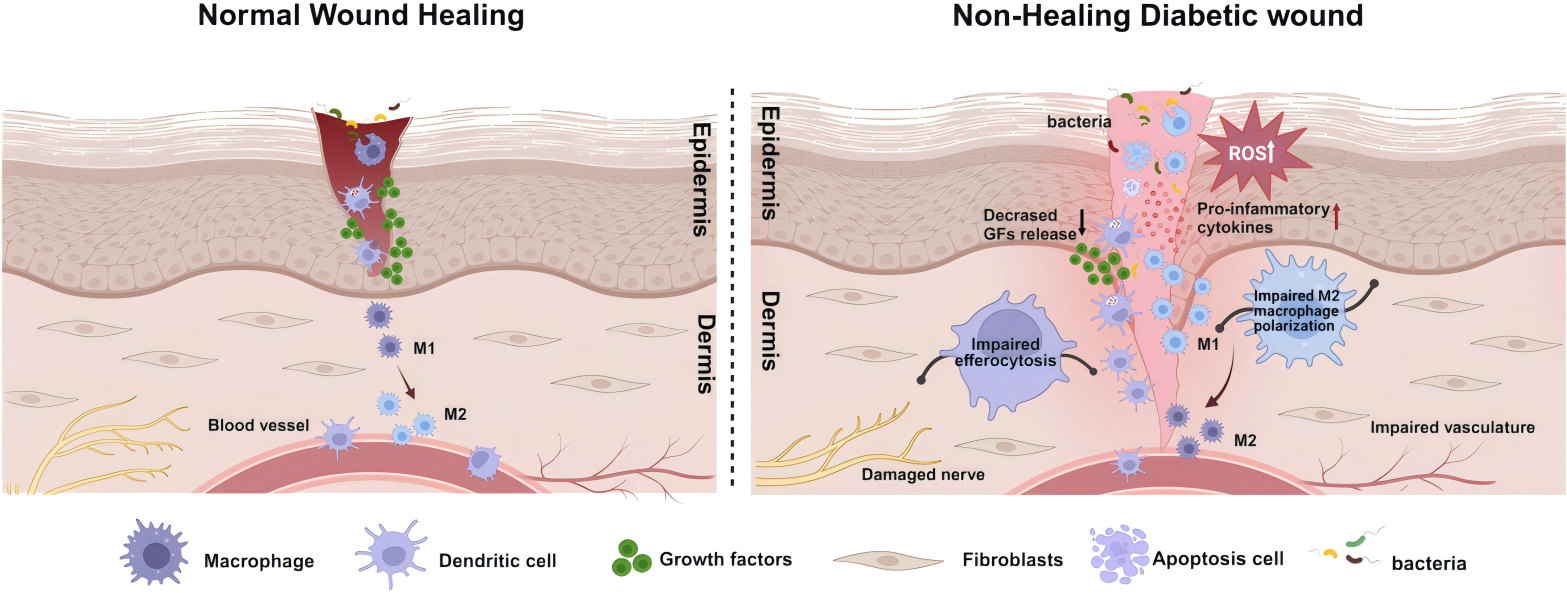
Schematic illustration of healing mechanisms in normal and non-healing diabetic wounds. The healing of wounds involves the coordinated and integrated interaction of various cell types, biomolecules, and the extracellular Matrix (ECM). Contrary to the healing process in normal wounds, in diabetic wounds, the prolonged and excessive inflammatory responses, along with a state of oxidative stress, are significant impediments to wound healing. This involves deficiencies in the immune system, particularly the absence of phagocytic cell efferocytosis, and an imbalance in antioxidative mechanisms, leading to the accumulation of reactive oxygen species (ROS) in the wound. In such circumstances, more inflammatory cells engage in the wound repair process, secreting pro-inflammatory factors. Concurrently, the decreased release of growth factors and an imbalance in macrophage polarization, further lead to damage to neural and vascular structures. These comprehensive outcomes subsequently affect the wound-healing process, ultimately resulting in a prolonged inflammatory phase and wounds that are challenging to heal. Created with BioRender.com.

Compared to normal skin, diabetic skin appears to have more inflammatory cell infiltration, edema, and less granulation tissue formation, leading to a decreased wound healing reserve capacity in diabetic wounds (53, 54). Abundant evidence suggests that the persistent excessive inflammatory immune response and oxidative stress state in wounds of diabetic patients are important aspects contributing to this adverse alteration (**Figure 2**). Systemic accumulation of advanced glycation end products (AGEs) due to hyperglycemia induces oxidative stress responses, damages skin and inflammatory cell functions, and increases the hardness of the extracellular matrix (ECM) (55, 56). Subsequent tissue damage leads the wound to a prolonged state of excessive inflammation, with a significant infiltration of neutrophils. The ROS and destructive enzymes produced by excessive inflammation perpetuate this adverse cycle (57). This oxidative stress impairs the healing, disrupts the regular order of overlapping healing stages, and ultimately promotes the emergence of a “chronic wound phenotype” (58, 59). Due to the high levels of inflammation and oxidative stress, the chronic wound environment provides perfect conditions for inducing senescent cells. Under the influence of a series of intrinsic and extrinsic factors, mitotic cells become senescent and non-proliferative. The senescence-associated secretory phenotype (SASP) of these cells, characterized by the secretion of proinflammatory cytokines (such as TNF-α, IL-6), chemokines, and tissue-degrading proteases (such as MMPs), further exacerbates the inflammatory environment and impedes wound healing (60). This hypersecretory phenotype disrupts the normal wound healing processes by maintaining a chronic inflammatory state, degrading extracellular matrix components, and potentially inducing senescence in neighboring cells (61), creating a self-perpetuating cycle of impaired healing.

### 3.1 Excessive inflammation response and immune system deficiencies

#### 3.1.1 Sustained excessive inflammatory response in diabetic wounds

In diabetic patients, the impairment of wound closure caused by hyperglycemia is closely associated with multiple factors, including atherosclerosis, dysfunction of skin cells, and peripheral neuropathy. These factors collectively increase the vulnerability of the skin and the risk of infection, thereby significantly delaying the healing process (62). Unlike acute wounds, the healing of diabetic wounds is characterized by a persistent excessive inflammatory phase, manifested by extensive infiltration of inflammatory cells such as neutrophils and macrophages (63–65). Concurrently, pro-inflammatory cytokines such as interleukin-1 (IL-1), interleukin-6 (IL-6), tumor necrosis factor alpha (TNF-α), and plasma C-reactive protein are continuously produced at the wound site (63, 64, 66, 67), coupled with heightened bacterial proliferation (64, 68). Under the influence of these inflammatory chemokines, pro-inflammatory cytokines such as β-defensins, keratinocyte chemotactic factors, and macrophage inflammatory proteins play a crucial role in inducing leukocyte aggregation at the wound site (12, 69, 70). However, in diabetic patients, the expression of inflammatory cytokines increases, β-defensin levels decrease, abnormal activation of sensor activation signals and transcription activators, and reduced activity of protein kinase B (PKB) and nuclear factor κB (NF-kappaB) (12, 69, 70), leading to a sustained excessive inflammatory response and severe impairment of the wound healing process. This persistent pro-inflammatory phenomenon has also been demonstrated in diabetic animal ulcer models as a significant impediment to wound healing (71).

#### 3.1.2 Immune deficiencies and cell dynamics in diabetic wound healing

Wound healing involves reactions from various immune systems and the participation of different immune cells. However, in diabetic patients, the impairment of these immune cell functions has been established (72). Impaired phagocytic activity and leukocyte dysfunction in obese and diabetic patients, compared to healthy individuals, may be related to elevated infection prevalence in this population (73). Chronic inflammation in diabetic wounds leads to T-cell accumulation and subsequently elevated levels of C-C motif chemokine receptor 4 and TNF-α, thereby significantly impacting the immune response and increasing susceptibility to opportunistic pathogens in wounds (74–76).

The immunological process of phagocytes clearing apoptotic cells from the injured area is known as efferocytosis, essential for tissue repair in skin wounds (77). Efferocytosis is carried out by “professional” phagocytes (such as monocytes, macrophages, and DCs) and “non-professional” phagocytes (such as epithelial cells) (78). After skin injury, DCs, neutrophils, and macrophages residing or recruited to the skin act as phagocytes, exerting an efferocytosis role to influence wound healing (5, 79, 80). Therefore, defects in efferocytosis are often associated with chronic inflammation of wounds and persistent inflammatory diseases (9, 81, 82). The presence of defects in the efferocytosis of phagocytes isolated from diabetic mice and humans in wounds, which exacerbate the inflammatory response, further confirms that the efferocytosis defects of phagocytes are a general characteristic of diabetes (9, 83).

### 3.2 The oxidative stress state in diabetic wounds

Several recent studies have revealed the crucial role of oxidative stress in diabetic wound healing. ROS are a class of molecules formed from oxygen, including free radicals and non-radical species such as singlet oxygen, superoxide anions, hydrogen peroxide, and hydroxyl radicals. Even low levels of ROS are beneficial against external damage (84). However, excess reactive nitrogen species and ROS in tissues can lead to oxidative stress. Oxidative stress and decreased antioxidant capacity cause redox imbalance and further damage cells and tissues, a significant reason for the non-healing of diabetic wounds (85). Redox imbalance is usually associated with an excessive accumulation of oxidative products and decreased activity of antioxidant enzymes. Clinical research has indicated that the high oxidative stress state in delayed healing diabetic wounds is related to hyperglycemia and tissue hypoxia. Antioxidant enzyme activities are also significantly reduced in patients with long-standing type 2 diabetes mellitus (T2DM) (86). Oxidative stress affects the normal wound-healing process and is closely related to neurovascular complications and ischemic hypoxic injury, which together account for the delayed healing of diabetic wounds.

#### 3.2.1 Excessive ROS production and antioxidant redox imbalance

Typically, cells control the excessive production of ROS through a range of antioxidant defense mechanisms. This includes enzymatic antioxidants such as GPX, catalase, and superoxide dismutase (SOD), as well as non-enzymatic antioxidants like glutathione, metal ion chelators, and vitamins. However, oxidative stress is triggered when the balance between ROS production and these antioxidants is disrupted. In diabetes, persistent hyperglycemia produces excessive ROS by increasing mitochondrial oxygen consumption, impairing mitochondrial function, or activating the evolutionarily conserved ROS-producing NADPH oxidase. In the microenvironment of chronic wounds, elevated ROS levels are associated with a range of harmful effects on wound healing, such as the production of AGEs (87). The levels of AGEs in the serum of diabetic patients and their receptors on the skin surface tend to be elevated (88, 89), and the interaction of these AGEs with endothelial cell surface receptors leads to excessive generation of ROS (90). The edge and center areas of diabetic wounds seem more prone to excessive oxidative stress and damage, which may further lead to poor wound healing (91, 92). Furthermore, AGEs impair wound contraction and prolong the inflammatory response, hampering the proliferation of the ECM (93). Elevated ROS levels impair endothelial cell function in wound repair, thus hindering angiogenesis (94). It also causes upregulation of intercellular adhesion molecule-1 expression, which promotes activated leukocyte exudation (95). These alterations inhibit keratinocyte migration, delaying the re-epithelialization of the wound (96). In terms of metalloproteinase regulation, ROS can lead to the inactivation of tissue inhibitors, promoting the formation of a proteolytic microenvironment in chronic wounds (97), and induce the expression of matrix metalloproteinases in fibroblasts, especially under the exposure to singlet oxygen (98), hydrogen peroxide (99), or hydroxyl radicals (100). Diabetic wounds, with their lower antioxidant reserves, provide favorable conditions for the accumulation of ROS. Studies have shown that compared to acute wounds, chronic wounds have significantly increased levels of SOD released by neutrophils (101) and have low levels of GSH (102). Additionally, the activation of oxidative stress response transcription factor Nrf2-related pathways has been shown to protect vascular endothelial cells from oxidative stress-induced damage, thereby promoting angiogenesis and accelerating wound healing in diabetic mice (103). However, in patients with type 2 diabetes and DFU, the circulating levels of Nrf2 and its downstream targets are significantly reduced (104, 105).

#### 3.2.2 Peripheral neuropathy and hypoxia in diabetic wounds

In diabetes, excessive oxidative stress can have detrimental effects on the structure, metabolism, and blood supply of peripheral nerves (106), leading to widespread deterioration in myelinated axons, Schwann cells, and sensory neurons located in the dorsal root ganglia (107). This may result in impaired nerve function and hypoxia. Increased ROS levels lead to DNA damage and polyadenosine ribose polymerase activation, a process that inhibits glucuronide dehydrogenase activity. This inhibition further triggers polyol and hexosamine pathways increases protein kinase C activity, promotes AGE formation, and activates apoptosis-related signaling pathways such as p38 MAPK and NF-kB. These changes lead to altered gene expression, apoptotic signaling, cytokine release, and microvascular dysfunction, ultimately resulting in impaired neurological function and the development of neuropathy (108). Additionally, oxidative stress, in conjunction with energy deficiency, contributes to axonal damage. Given that axons are rich in mitochondria, providing a direct supply of neural energy, a lack of ATP supply could result in the loss of ability to transport along the axon, exacerbating axonal injury and leading to diabetic neuropathy. This phenomenon is further aggravated under oxidative stress, triggering axonal degeneration or apoptosis (109). Studies show that at least half of diabetic patients develop clinically significant peripheral neuropathy (110, 111). This peripheral neuropathy can induce sensory, motor, and autonomic nerve function disorders, leading to delayed wound healing (15). Sensory nerve dysfunction reduces diabetic ulcer patients’ awareness of wounds (112), leading to diminished or loss of skin protection (57), which represents a significant threat to diabetic ulcer wound regeneration (15). By increasing plantar pressure, motor neuropathy directly damages tissues, leading to the occlusion of plantar capillaries, local tissue ischemia, and destruction (113). Autonomic neuropathy caused by diabetes manifests as abnormalities in sweat gland function, resulting in abnormal temperature regulation, reduced skin sweating, and dry, cracked skin. These changes further compromise skin integrity, making the wound more susceptible to infection (114). Moreover, dysfunction of the peripheral sympathetic nerves may lead to dysregulation of vascular regulation and abnormal arteriovenous shunting, potentially causing abnormalities in skin blood flow and microcirculatory dysfunction, thus causing local blood circulation distribution disorders and insufficient nutritive capillary blood supply (57). This circulatory disorder, causing insufficient oxygen and nutrient supply to diabetic wounds, is further exacerbated under the influence of hemoglobin glycation (69).

#### 3.2.3 Microvascular complications and ischemia in diabetic wounds

Microvessels serve as natural conduits for the transport of blood and immune cells, and they supply nutrients to the skin of the lower limbs (115). In diabetic wounds, complications in these microvessels exacerbate ischemia and hypoxia at the wound site, posing challenges to normal healing processes. Diabetic microvascular complications arise from a complex pathology involving microangiopathy and macroangiopathy, driven by processes such as non-enzymatic glycation, oxidative stress, inflammation, endothelial dysfunction, and a hypercoagulable state (116, 117). These factors collectively lead to endothelial cell damage and dysfunction. Unlike other cell types, such as skeletal muscle cells, adipocytes, and hepatocytes, endothelial cells have a reduced capacity to decrease glucose uptake in response to elevated extracellular glucose levels, leading to hyperglycemia within these cells and consequent oxidative stress and damage (118). Excess ROS leads to endothelial dysfunction through various mechanisms, including activation of biochemical pathways such as AGE/RAGE, polyols, hexosamine, and protein kinase C. These pathways trigger oxidative stress, leading to inflammation, reduced nitric oxide, pericyte degeneration, endothelial hyperplasia, basement membrane thickening, impaired vasorelaxation, and increased procoagulant activity, thereby contributing to the progression of diabetic microangiopathy (119–122). Structural changes in the microvasculature of diabetic wounds impede the delivery of nutrients and activated leukocytes, increasing tissue susceptibility to infection and exacerbating inflammation reaction, then promoting the development and progression of diabetic wounds (57). Additionally, these structural changes increase the likelihood of microvascular occlusions, further exacerbating local ischemia and hypoxia in diabetic wounds (57). MicroRNAs, such as miR-210 induced by hypoxia-inducible factor-1 alpha in ischemic wounds, play significant roles in diabetic microvascular complications by inhibiting keratinocyte proliferation and hindering wound closure (123).

## 4 Mechanism of SLC7A11 in regulating diabetic wound healing

### 4.1 SLC7A11 and efferocytosis in diabetic wound healing

**Figure 3:**
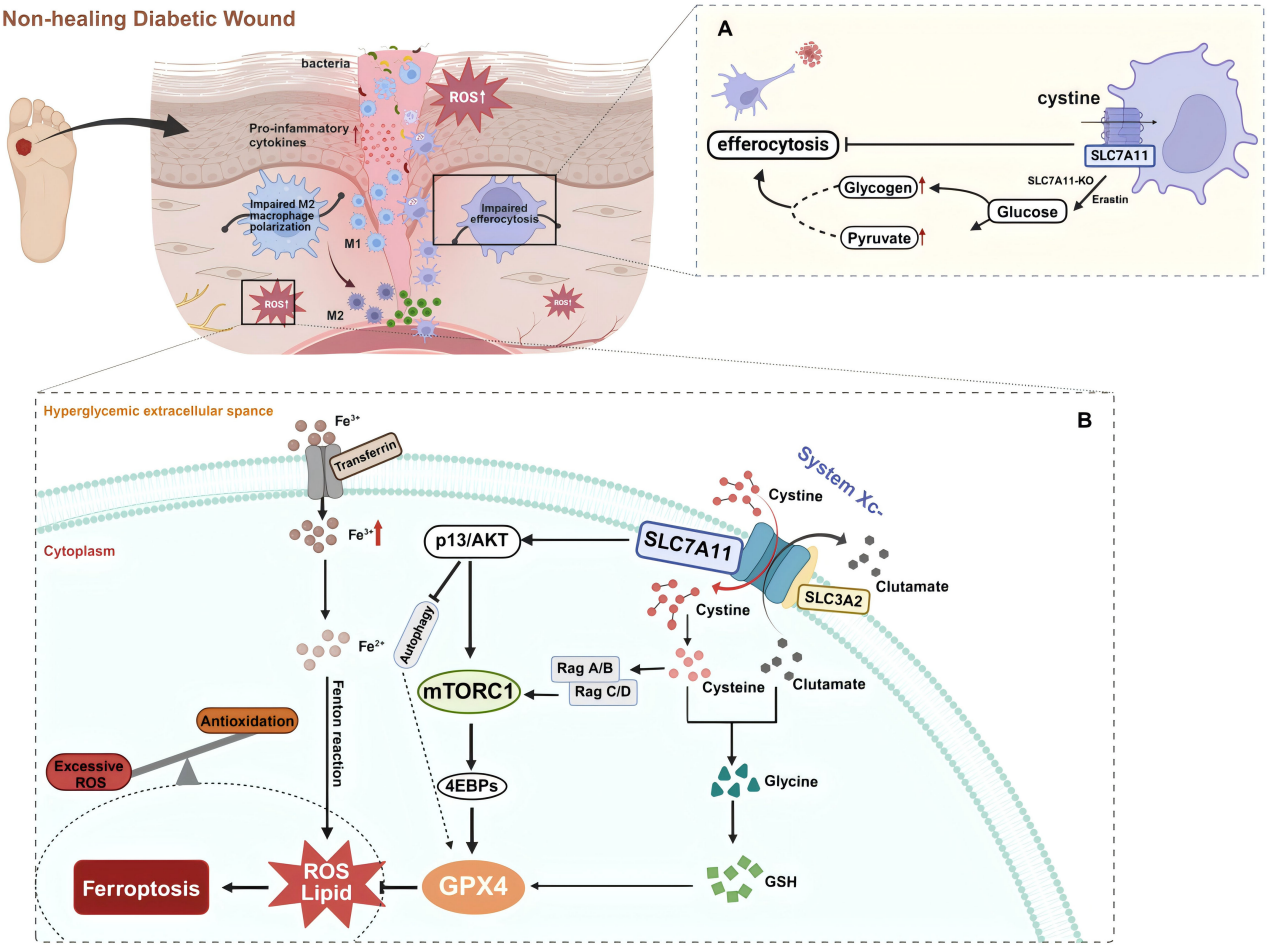
**(A)** SLC7A11 acts as a brake on dendritic cell (DC) efferocytosis. Efferocytosis in DCs is enhanced via glycogenolysis with the inhibition or knockout of SLC7A11 during diabetic wound healing. **(B)** SLC7A11 is a multi-pass transmembrane protein that mediates the activity of the cystine/glutamate antiporter, system Xc-. SLC3A2 is a companion protein that maintains the stability and proper membrane localization of SLC7A11. Together, they form the system Xc- via an extracellular covalent disulfide bond and operate in a 1:1 ratio to export intracellular glutamate and import extracellular cystine, which provides cysteine for glutathione synthesis. Extracellular cystine is imported into the cell via SLC7A11 and then reduced to cysteine. Cysteine combines with glutamate, and in the presence of enzymatic addition of a glycine molecule, produces glutathione (GSH). GSH neutralizes and clears reactive oxygen species (ROS), protecting cells from oxidative damage. Ferroptosis is a form of non-apoptotic cell death that depends on lipid oxidation and the accumulation of intracellular iron. Glutathione peroxidase 4 (GPX4) uses GSH to reduce lipid hydroperoxides to lipid alcohols, thereby inhibiting ferroptosis. The cystine uptake mediated by SLC7A11 and PI3K/AKT signaling promotes the activation of mTORC1, which through the Rag-mTORC1-4EBP signaling axis, enhances GPX4 protein synthesis. It also upregulates the SREBP1-SCD1 axis to increase MUFA synthesis and inhibits autophagy, thereby suppressing lipid peroxidation and ferroptosis. In the context of diabetic wounds, these metabolic balancing processes are disrupted. Trivalent iron (Fe3+) binds to transferrin and is imported into cells via the membrane protein transferrin receptor 1. Fe3+ is then reduced to Fe2+ by a reductase. Finally, excessive intracellular iron mediated by high glucose levels generates ROS through the Fenton reaction, leading to ferroptosis. Created with BioRender.com.

In diabetic wounds, continuous cell death triggers inflammatory responses, disrupting the tissue repair process (78, 82, 124). During this process, phagocytic cells such as macrophages clear the dead cells from the wound through a process known as efferocytosis to mitigate these negative effects. Among these, DCs are a heterogeneous group of professional phagocytes capable of recognizing and engulfing apoptotic cells. DCs are essential in maintaining tissue homeostasis by regulating innate and adaptive immune responses. Co-stimulatory molecules such as CD80, CD86, and MHC-II are essential for DCs maturation and subsequent activation of native CD4 T cells. Their deficiency may lead to T cell immunosuppression or tolerance, whereas effective efferocytosis by DCs suppresses the immune responses to self-antigens. Meanwhile, abundant evidence highlights the favorable aspects of efferocytosis by DCs in diabetic wound healing. After phagocytosing infected apoptotic cells, DCs release large amounts of cyclooxygenase-2, PGE2, IL-10, and TGF-β, which contribute to balancing inflammatory responses and promoting tissue repair (125–127). Recent research has revealed that SLC7A11 primarily regulates efferocytosis in dendritic cells within diabetic mouse skin wounds, acting as a critical regulatory factor in diabetic wound healing. DCs treated with erastin or SLC7A11-KO show increased aerobic glycolysis, which fulfills the bioenergetic demands of efferocytosis. Pharmacologically inhibiting the function of SLC7A11 significantly enhances wound healing in diabetic mice (5). Experiments indicate that in db/db mice, which are prone to diabetes, reduced efferocytosis and lower levels of GDF15 are associated with high expression of SLC7A11 (5). Therefore, inhibiting SLC7A11 in wounds can promote diabetic wound healing through at least two mechanisms: improving cellular efferocytosis and enhancing the release of factors such as GDF15 by efferocytic cells (**Figure 3A**).

### 4.2 The role of SLC7A11 in oxidative stress and ferroptosis in diabetic wounds

Among the multiple pathological mechanisms leading to abnormalities in diabetic wound healing, there is gradually a widespread concern about the impairment of wound healing by hyperglycemia-induced ROS accumulation and oxidative stress. Ferroptosis, an iron-dependent form of non-apoptotic cell death typically characterized by the accumulation of lipid ROS (14), has been proven to be closely associated with the pathological process of diabetic wound healing (17). Ferroptosis damages wound healing in diabetic patients by affecting the pathology of ROS and lipid peroxidation products (15). In a high-glucose environment, the increased ROS, lipid peroxidation, and ferroptosis-related proteins in fibroblasts and endothelial cells lead to reduced viability and migration rates, whereas the ferroptosis inhibitor Fer-1 can significantly alleviate these adverse effects (13). An animal study showed that pharmacological inhibition of ferroptosis reduces the expression of inflammatory and oxidative stress markers in diabetic rat wounds and promotes wound healing by activating the PI3K/AKT pathway (13). Thus, effective inhibition of ferroptosis may positively affect diabetic wound healing. Notably, SLC7A11 plays a vital role in the ferroptosis mechanism through its unique function in mediating cystine/glutamate exchange (**Figure 3B**). SLC7A11 promotes cellular cystine uptake, providing the necessary precursors for synthesizing GSH, which is crucial for detoxifying cytotoxic lipid peroxides by glutathione peroxidase 4 (GPX4). By utilizing GSH, GPX4 reduces lipid peroxides to lipid alcohols, thereby inhibiting the occurrence of ferroptosis (128). In diabetic conditions, sustained high glucose-induced oxidative stress results in the downregulation of SLC7A11 expression, which decreases intracellular cystine levels. This reduction leads to depleted GSH biosynthesis and inhibits GPX4 activity, ultimately triggering ferroptosis (129). Therefore, activating SLC7A11 to import cystine and promote glutathione synthesis can effectively prevent the accumulation of lipid peroxides and the occurrence of ferroptosis, protecting diabetic wound healing (20).

### 4.3 Influence of SLC7A11 on insulin secretion

**Figure 4:**
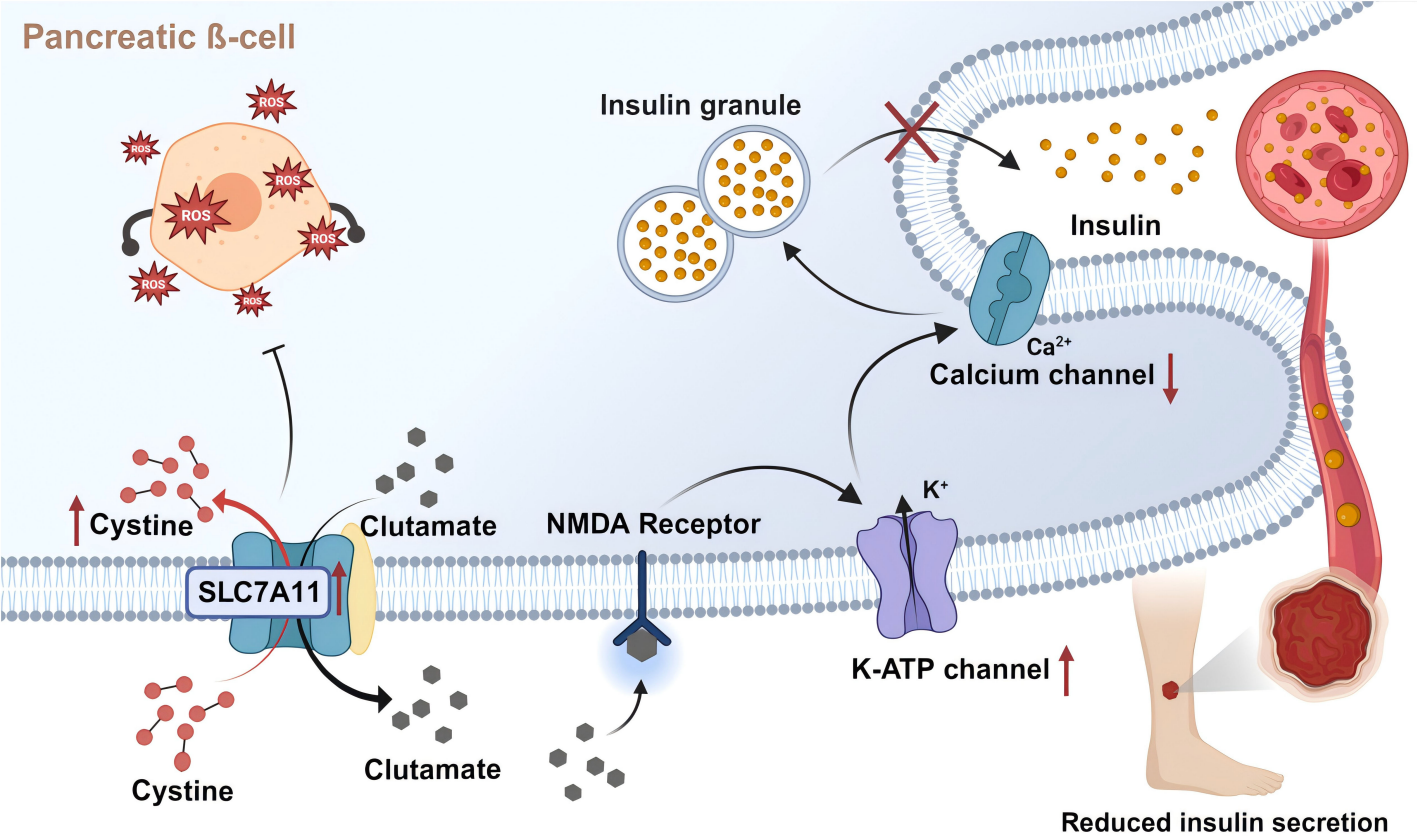
SLC7A11 transports glutamate to regulate insulin secretion. Pancreatic ß-cells partially release glutamate extracellularly via system Xc- (SLC7A11). Extracellular glutamate activates NMDA receptors on ß-cells, inducing reactivation of the off K-ATP channel. This leads to repolarization of islet cells and closure of calcium channels, reducing insulin secretion rate. In addition, SLC7A11 imports cystine and synthesizes GSH, thereby inhibiting the impairment of islet viability by oxidative stress and ferroptosis. Created with BioRender.com.

In T2DM, disruption of the insulin signaling pathway is a known pathological factor leading to impaired wound healing (130). Reduction in the number of cell surface insulin receptors, the impediments of PI3K activation, and subsequent signal transmission within the insulin signaling pathway contribute to ineffective insulin work, thereby developing insulin resistance (131). In addition, insulin regulates the differentiation of skin keratin-forming cells and glucose transport through IR-related signals (132). Expression of insulin receptor, insulin receptor substrate 1 (IRS-1), insulin receptor substrate 2 (IRS-2), Src Homology 2 Domain Containing, Extracellular Signal-Regulated Kinase (ERK) and AKT are increased in healing wound tissues compared to intact skin. In contrast, these pathways are weakened in the injured skin of diabetic rats. Topical application of insulin normalizes wound healing time in diabetic animals, subsequently reversing defective insulin signal transduction (133). In pancreatic β-cells, insulin secretion is tightly associated with the glucose metabolism process and its various by-products, ranging from ATP and glutamate (19) to ROS (134). This relatively unrestricted glucose metabolic pathway renders β-cells particularly sensitive to oxidative stress induced by hyperglycemia (135, 136). The Slc7a11 gene is upregulated to provide sufficient cysteine to cope with the inflammatory response and alleviate the increase in ROS production, mitigating cellular damage (137). Studies indicate that constitutive deficiency of system Xc- results in approximately a threefold decrease in GSH levels in the islets, characteristic upregulation of Chac1 due to cysteine deficiency, and reduced β-cell-specific gene expression and increased markers of endoplasmic reticulum stress, thus diminishing insulin secretion both *in vitro* and *in vivo* (137). Furthermore, ferroptosis induced by the inhibition of system Xc- with erastin has been shown to impair islet viability (138). Even though intracellular glutamate enhances insulin secretion, extracellular glutamate might activate ionic receptors, thereby slowing insulin exocytosis (**Figure 4**). This process is regulated by system Xc- (19), presenting a double-edged sword for pancreatic β-cells.

### 4.4 SLC7A11 and mTOR-related pathways

The mTOR pathways play a core role in maintaining cellular and physiological homeostasis, closely associated with various growth disorders such as obesity and T2DM, and regulate insulin sensitivity and glucose homeostasis (139). PI3K also activates another serine/threonine kinase, AKT, a key signaling hub for multiple cellular functions. Activation of AKT affects a series of downstream pathways, including cell survival, proliferation, aging, apoptosis, and angiogenesis (140, 141). Studies show that reduced activity of the PI3K/AKT signaling pathway weakens cellular responses such as insulin uptake, thus promoting the development of DM (140). In DM, multiple signaling pathways, including epidermal growth factor receptor (EGFR)/PI3K/AKT and extracellular signal regulated kinases (ERKs), are affected and activate their downstream Bcl2-associated death signaling, increasing apoptosis and decreasing proliferation, then impeding wound healing (142). Further research highlights the prominent role of SLC7A11 in regulating these changes. It was shown that SLC7A11-mediated uptake of cystine and PI3K/AKT signaling activates mTORC1, at least partly through the Rag-mTORC1-4EBP signaling axis to promote GPX4 protein synthesis (18). This activation enhances MUFA synthesis and inhibits autophagy by upregulating the SCD1-SREBP1 axis, thus preventing lipid peroxidation and ferroptosis (143). This mechanism differs from the conventional model in which SLC7A11 regulates GPX4 and inhibits ferroptosis via cysteine-mediated GSH synthesis, forming the SLC7A11-GSH-GPX4 axis. Additionally, the interaction between mTOR signaling targets and GPX4 regulates autophagy-dependent ferroptosis in human pancreatic cancer cells (144). Notably, SLC7A11 can modulate selenium uptake and directly enhance selenium-dependent GPX4 protein levels (145, 146). However, this mechanism seems to promote GPX4 synthesis by a cellular sensing mechanism independent of mTORC1 mediation (147).

## 5 The role of SLC7A11 in the treatment strategies for diabetic wounds

### 5.1 Immune regulation strategies in diabetic wound related to SLC7A11

Diabetic wounds are primarily characterized by persistent stagnation in a non-productive inflammatory stage, leading to compromised formation and consolidation of mature granulation tissue (148, 149). This prolonged inflammatory phase, unrelated to the successful control of local infections, suggests that the susceptibility of diabetic wounds to infections (150–152) might stem from a primary defect in innate immune response mechanisms (153, 154). During the healing process, the plasticity of macrophages allows them to transition from an initial pro-inflammatory phenotype (M1) to an anti-inflammatory phenotype (M2), facilitating tissue repair (155, 156). However, in non-healing diabetic wounds, macrophage dysfunction leads to a pronounced pro-inflammatory state (157). The reduced capability of macrophages in diabetic patients to perform efferocytosis may also contribute to the decreased prevalence of the M2 phenotype, partially promoting an increase in M1 macrophages (158). Meanwhile, hyperglycemia serves as a primary trigger for excessive inflammatory responses. Research indicates that hyperglycemia and its related factors, such as AGEs, TNF-α, and other pro-inflammatory signaling proteins, exhibit significant cytotoxicity detrimental to the recovery of wound tissues (58, 159). AGEs promote macrophage polarization to the M1 phenotype through autophagy activation (160). Due to their heightened response to inflammatory stimuli, macrophages release more inflammatory factors and exhibit reduced phagocytosis of pathogens and apoptotic cells, thus impairing pathogen clearance (161). The PI3K-AKT-mTOR signaling pathway mediates the perception of environmental and metabolic cues, influencing macrophage polarization in a complicated and not yet fully defined manner (162). Amino acids and other potential metabolic inputs into the Akt-mTORC1 axis align M2 activation with metabolic status (163). For example, glutamine metabolism promotes the production of UDP-GlcNAC, a crucial modifier of various M2 markers (164). Oxidative metabolism has also been shown to correlate with M2 activation, even though specific mechanisms remain unclear. These factors collectively contribute to the pro-inflammatory, pro-oxidative, and pro-degradative environment within diabetic wounds (165). Therefore, targeting SLC7A11 to modulate the immune and inflammatory responses in diabetic wounds presents a promising therapeutic strategy. Ginsenoside Rg5, a rare ginsenoside, exerts hypoglycemic effects by improving insulin resistance and mitochondrial biogenesis in diabetic mice (166). Recent studies indicate that ginsenoside Rg5 can inhibit the expression and activity of SLC7A11 through physical binding, essentially counteracting SLC7A11’s negative regulation of glycolysis, which facilitates the efferocytosis by DCs (**Table 1**) (167).

**Table 1:** Immune modulation and antioxidant therapeutic strategies related to SLC7A11.

Medication	Modification in SLC7A11	Conventional Roles	Reference
Ginsenoside Rg5	Suppression of SLC7A11 expression and activity	Hypoglycemic effects	(167)
Xanthohumol	Upregulate SLC7A11 expression	Alleviate oxidative damage and accelerate diabetic wound healing	(168)
Liraglutide	UpregulateSLC7A11 expression	Treatment of obesity and diabetes	(171)
Bee venom	Upregulate SLC7A11 expression	Enhance wound closure in diabetic mice	(169)
Canagliflozin	Upregulate xCT expression	Hypoglycemic effects	(170)
Quercetin	Repress SLC7A11 expression	Reversal of ferroptosis in lungs	(172)
Glab	Upregulate SLC7A11 activity	Ameliorate diabetes	(174)
Astaxanthin	Upregulate xCT expression	utilized in the treatment of diabetes and cardiovascular diseases.	(175)
Cryptochlorogenic acid	Upregulate xCT expression	Diabetes treatment	(176)
Zeaxanthin	Upregulate SLC7A11 expression	Inhibition of ferroptosis to intervene in nonalcoholic fatty liver disease	(177)
Sulforaphane	Upregulate SLC7A11 expression	Inhibition of ferroptosis in cardiac cells of mice with diabetic cardiomyopathy	(178)
RSV	Upregulate xCT expression	Enhancement of the osteogenic potential of human dental pulp stromal cells	(179)
Tuberostemonine	Upregulate SLC7A11 expression	Inhibition of ferroptosis thereby alleviating pulmonary fibrosis	(173)

### 5.2 Antioxidant strategies in diabetic wound treatment targeted SLC7A11

SLC7A11 promotes cystine uptake and glutathione biosynthesis, thus preventing oxidative stress and ferroptosis (20), constituting one of the primary antioxidative mechanisms in diabetic wounds. Consequently, pharmacological modulation of SLC7A11 to enhance antioxidation represents a promising therapeutic strategy, as illustrated in **Table 1**. Xanthohumol, a prenylated dietary flavonoid derived from hops, has been shown to mitigate oxidative damage and accelerate diabetic wound healing through NRF2 activation, positioning it as a potential nutritional supplement and candidate drug for treating diabetic skin ulcers (168). Similarly, bee venom significantly enhances wound closure in diabetic mice by increasing the expression of collagen and β-defensin 2 and restoring the levels of Angiopoietin-1 and NRF2 (the agonist ligands of Tie-2), thereby enhancing downstream signaling of Tie-2 (169). Traditional antidiabetic drugs target SLC7A11 to inhibit ferroptosis and exert antioxidative effects. Canagliflozin, an antidiabetic drug, benefits cardiovascular health in a diabetic cardiomyopathy model by regulating cardiac iron balance and the system Xc-/GSH/GPX4 axis to inhibit ferroptosis (170). Liraglutide, used for treating obesity and diabetes, inhibits ferroptosis in db/db mice by enhancing the expression of the SLC7A11 and NRF2/HO-1/GPX4 signaling pathways, reducing ferritin deposition and lowering intracellular lipid ROS (171).

Extensive research indicates that bioactive compounds extracted from animals and plants effectively regulate the expression and activity of SLC7A11. Quercetin, a natural dietary flavonoid, improves the levels of the antioxidative protein GPX4 and SLC7A11 in the lung tissues of patients with neutrophilic airway inflammation, reversing lung ferroptosis (172). Components extracted from the medicinal plant Stemona, such as Tuberostemonine, alleviate pulmonary fibrosis by regulating SLC7A11, GPX4, and GSH, reducing iron and ROS accumulation (173). The well-known diabetes-ameliorating effects of Glabridin (Glab) from licorice have been demonstrated. A recent studies show that Glab protects diabetic rat kidneys by enhancing SOD and GSH activities, increasing the expression of SLC7A11, GPX4, and SLC3A2, and lowering malondialdehyde and iron concentrations as well as transferrin receptor 1 expression, thereby inhibiting ferroptosis (174). Astaxanthin, a natural ketocarotenoid pigment, mitigates IL-1β-induced chondrocyte ferroptosis by regulating intracellular ROS, iron, and mitochondrial iron levels and by inhibiting the expression of SLC7A11, GPX4, and ferritin, providing relief for osteoarthritis (175). Cryptochlorogenic acid from mulberry leaves activates the system Xc-/GPX4/NRF2 pathway. It inhibits nuclear receptor coactivator 4, improving blood glucose levels, iron content, lipid peroxidation, and pancreatic damage in a diabetes model (176). Additionally, some drugs regulate SLC7A11 in a p53-dependent manner, such as zeaxanthin, which regulates GPX4, SLC7A11, and other targets by downregulating p53 expression, reducing cellular lipid peroxidation and intervening in the development of non-alcoholic fatty liver disease (177). The medications mentioned have been demonstrated to mitigate oxidative stress and ferroptosis in disease by modulating the expression and activity of SLC7A11 and its downstream molecules, thereby alleviating the progression of the disease. This molecular mechanism of action, achieved based on targeting SLC7A11, provides a potential therapeutic strategy for treating diabetic wounds.

## 6 Summaries and outlook

**Figure 5:**
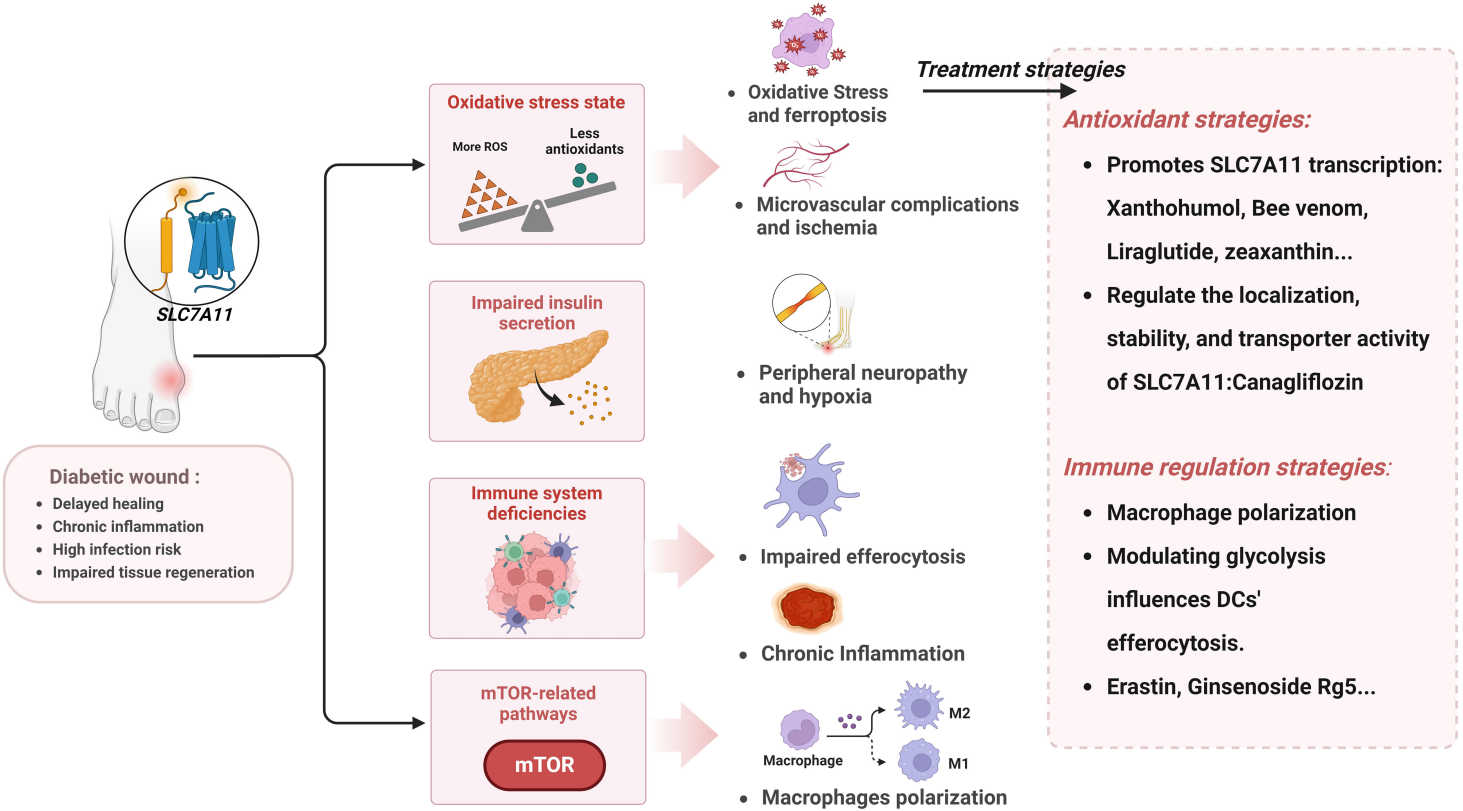
The pathological mechanisms in diabetic wound related to SLC7A11 and prospective therapeutic interventions. Created with BioRender.com.

Diabetes is increasingly prevalent on a global scale. Particularly diabetic wound complications, which are both common and severe, often leading to amputation and even death. It thus constitutes a significant burden on the global economy and public health systems. In this context, developing effective therapeutic agents to improve the outcome of diabetic wounds is particularly urgent. In recent years, the roles of oxidative stress and aberrant inflammatory regulation in the healing process of diabetic wounds have attracted increasing attention. Compared to normal wound healing, the inflammatory response in diabetic wounds is more intense and persistent, and oxidative stress is relatively increased, leading to prolonged healing processes and impaired tissue remodeling. Based on the close relationship between SLC7A11 and the above pathomechanisms, this article reviews the pathological mechanisms of diabetic wounds, focusing particularly on the abnormalities in inflammatory responses and oxidative balance, and explores the critical role of SLC7A11 in regulating the healing process of diabetic wounds (**Figure 5**). First, SLC7A11 regulates efferocytosis to normalize the immune response at the wound site, although excessive efferocytosis can also exacerbate inflammation. Then, through the system Xc- exchange mechanism, cells can export glutamate and absorb cystine, subsequently synthesizing GSH to maintain oxidative balance. Properly regulating the activity of SLC7A11 can effectively counteract the accumulation of ROS and oxidative stress caused by the hyperglycemic environment, thus preventing ferroptosis in the wound. Additionally, SLC7A11 is closely associated with insulin secretion and the mTOR signaling pathway. Theoretically, therapeutic strategies based on SLC7A11 demonstrate a highly promising potential. Currently, drugs targeting SLC7A11 are being developed, and they have shown potential in promoting diabetic wound treatment *in vitro* experiments and animal models.

Although this review delves into the critical role of SLC7A11 in diabetic wound healing, it must be acknowledged that diabetic wound healing is a multifactorial-driven complex process involving various aspects, such as angiogenesis, microbial infection, and diabetes-induced systemic complications. These areas still require further systematic investigation. It is important to note that while SLC7A11 inhibitors, such as Erastin, have shown some efficacy in cancer research, their targeted application in diabetic wounds still needs to be confirmed. The inhibition of SLC7A11 activity, while potentially enhancing immune cell infiltration within the wound, may also exacerbate oxidative stress, leading to increased ferritin deposition and adverse outcomes. Therefore, precise regulation of SLC7A11 is crucial in addressing the complexity and variability of therapeutic interventions. Although SLC7A11 presents a promising target for improving diabetic wound healing, developing a comprehensive and effective therapeutic strategy requires a broader context that integrates other relevant mechanisms and pathways. A deep understanding of these complex biological processes is essential to translating these research advances into clinical interventions and ultimately improving patient outcomes.
